# Socioeconomic Inequalities and Factors Associated with the Use of Modern Contraceptive Methods in Women of Childbearing Age in Ecuador, 2018

**DOI:** 10.3390/healthcare11162293

**Published:** 2023-08-14

**Authors:** Sandra Callata-Cardenas, Fátima Milagros del Rosario Peña-Cerna, Akram Hernández-Vásquez, Diego Azañedo

**Affiliations:** 1 Faculty of Health Sciences, Universidad Científica del Sur, Lima 15067, Peru100036930@cientifica.edu.pe (F.M.d.R.P.-C.);; 2 Centro de Excelencia en Investigaciones Económicas y Sociales en Salud, Vicerrectorado de Investigación, Universidad San Ignacio de Loyola, Lima 15046, Peru

**Keywords:** female contraception, contraceptive use, social determinants of health, health care disparities, Ecuador

## Abstract

The objective of this study was to determine the socioeconomic inequalities and factors associated with the use of modern contraceptive methods (MCM) in the population of sexually active women of childbearing age in Ecuador. This was an analytical observational study, based on a secondary data analysis of the 2018 National Health and Nutrition Survey (ENSANUT). Information on 19,106 sexually active, married, or cohabiting women between the ages of 15 and 49 were included. Concentration curves (CC) and Erreygers concentration indices (ECI) were calculated, taking into account the use of MCM as the dependent variable and the wealth index as the independent variable. Crude and adjusted prevalence ratios with 95% confidence intervals were calculated using generalized linear models of the Poisson family. We found that 92.8% of the women surveyed used some type of MCM in the last month. A higher educational level presented a significant pro-rich concentration in the use of MCM (EIC: 0.05; *p* = 0.004). On the other hand, women belonging to the age group of 20 to 29 years (ECI: −0.027; *p* = 0.027), women with no job (ECI: −0.025; *p* = 0.004), and non-indigenous women (EIC: −0.031; *p* < 0.001), presented a pro-poor concentration. Factors significantly associated with MCM use were age, marital status, occupation, parity, ethnicity, area of residence, and living on the coast. In Ecuador, there are socioeconomic inequalities at different levels of population subgroups in women of childbearing age. Measures to promote the use of MCM are required, focusing on groups that present inequality, taking into account the factors associated with their use.

## 1. Introduction

One of the key indicators of Sustainable Development Goal 3.7 is increasing the proportion of women of reproductive age able to meet their family planning needs through modern methods by 2030 [1]. According to the World Health Organization, 74 million women in low- and middle-income countries have unplanned pregnancies annually, which are associated with 25 million unsafe abortions and almost 47,000 maternal deaths [2]. It is also estimated that, worldwide, more than 250 million women of reproductive age have an unmet need for contraceptive methods (CM); i.e., they wish to interrupt or delay childbearing for at least 2 years, but do not use any CM [3]. Under this definition, Latin America and the Caribbean (LAC) has a 22% unmet need for modern contraceptive methods (MCM) [3]. Furthermore, in Ecuador, in 2018, almost 30% of women of childbearing age (15–49 years) did not use MCM (including male sterilization or vasectomy; female sterilization or ligation; implants; contraceptive injection; contraceptive pills; intrauterine device; male or female condom; and emergency contraceptive pills) [4]. Consequently, there is an urgent need to address this problem to reduce the incidence of unwanted pregnancies and their social, economic, and maternal and fetal morbidity and mortality consequences, especially in LAC countries [5].

Several studies have reported the presence of inequalities in the use of MCM according to certain population characteristics [6,7,8,9,10,11]. Thus, a study that included 11 low- and middle-income countries in the African and Asian regions identified inequalities in the satisfied demand for MCM in favor of women within the highest wealth quintile, those who were older, and women with higher levels of education [11]. Likewise, a study in LAC identified wealth-related inequalities in favor of the highest quintile in the use of MCM, which were greater in Guatemala, Bolivia, and Suriname [7]. On the other hand, the literature reports that factors associated with satisfied demand for MCM include women’s age, religion, occupational status, educational level, wealth quintile, knowledge about modern methods, number of children, marital status, and having experienced unwanted pregnancies, among others [8,9,10]. Having this information is relevant for LAC countries, due to the high heterogeneity in the distribution of these characteristics, which can determine patterns of adoption of public health measures among the population, such as the use of CM, as well as assess the progress of inequalities over time.

To date, there have been no studies with updated information on the evaluation of socioeconomic inequalities and factors associated with the use of MCMs in the population of sexually active women of childbearing age in Ecuador. One study reported that Ecuador was identified as a country with intermediate inequality in the prevalence of CM use (a difference of approximately 25 percentage points in prevalence between the highest and lowest wealth quintile) in 2004 [7]. Another study, that evaluated inequalities in the coverage of reproductive, maternal, neonatal, and child health interventions, as well as the use of MCM, using information from a national survey performed in 2012, determined that, after adjusting for the wealth index, educational level and area of residence, indigenous women had a lower probability (prevalence ratio [PR] = 0.76; 95% confidence interval [CI]: 0.7–0.8) of using MCM compared to self-identified non-indigenous women [12]. In 2018, the results of the National Health and Nutrition Survey (ENSANUT-2018) reported that the percentage of non-use of MCM by Ecuadorian women of reproductive age was almost 8%; however, differences were identified at the level of the region of residence, being higher in the Amazon area (11.4%), and lower in the insular area (5.1%) [13]. These figures show that the use of MCM by Ecuadorian women of childbearing age could be influenced by sociodemographic characteristics; however, no information in this respect has been published to date.

The ENSANUT-2018 collected information on the health and reproductive conditions of women of childbearing age considering the geographic, demographic, cultural, ethnic, social, and economic diversity of the country [14]. In this sense, the objective of this study was to determine the socioeconomic inequalities and factors associated with the use of MCM in the population of sexually active women of childbearing age in Ecuador. The results of this study will guide the design of future research and interventions that will allow authorities to generate public health strategies to promote the use of MCM and improve their timely access.

## 2. Materials and Methods

### 2.1. Study Design and Data Sources

We performed an analytical cross-sectional study based on a secondary analysis of the data obtained from the ENSANUT-2018 conducted in Ecuador by the National Institute of Statistics and Census (INEC). This survey is conducted every five years and its main objective is to generate indicators on the main problems of the Ecuadorian population and its health situation to evaluate and develop public policies on health and nutrition issues [15].

The databases, technical documents, manuals, and description of variables are in the public domain, and are made available by the National Institute of Statistics and Census (INEC): (https://anda.inec.gob.ec/anda/index.php/catalog/891/sampling) (accessed on 14 November 2022).

### 2.2. Population and Sample

The target population of the ENSANUT-2018 was all household members, and it was specifically aimed at collecting information on women of childbearing age from 10 to 49 years old, children under 5 years old, men 12 years old and older, and children 5 to 17 years old. The ENSANUT-2018 used a two-stage, stratified probability sampling method that included a total of 2591 clusters and 46,638 households at the national level with geographic coverage of the 24 provinces of Ecuador [13].

In the first stage, a stratified sample of primary sampling units was selected (clusters of private dwellings according to the Political Administrative Division defined in the Geographic Statistical Classifier) (https://www.ecuadorencifras.gob.ec/documentos/web-inec/Geografia_Estadistica/Micrositio_geoportal/index.html#clasificador-geog-dpa) (accessed on 14 November 2022). In the second stage, a variable number of private dwellings were randomly selected (18 on average per primary sampling unit), and the research unit was the households of the selected dwellings and their usual residents of eligible households (persons who had stayed overnight the night before the survey) [14].

The sample for the present study included 19,106 sexually active women aged 15–49 years who were married or had a partner at the time of the survey and who had complete information on the variables of interest for the study (expanded population: 1,793,705).

### 2.3. Variables

#### 2.3.1. Dependent Variable

The dependent variable was the use of MCM in the last month according to the definition adopted by ENSANUT-2018, which include male sterilization or vasectomy; female sterilization or ligation; implant or contraceptive injection; contraceptive pills; intrauterine device; female or male condom; and emergency contraceptive pills [13]. This variable was constructed from the information obtained from the question “In the last month did you use or not use any contraceptive method”. This variable is a dichotomous categorical variable (Yes used/Not used).

#### 2.3.2. Independent Variable for the Analysis of Inequalities

The independent variable wealth index was constructed from information on household characteristics and assets obtained from the household form of the ENSANUT-2018 [15]. The wealth index is a composite measure of a household’s cumulative standard of living, the methodology of which is widely used internationally in national health surveys [16].

For estimation of the wealth index, the variables described in a previous study on the construction of a wealth index with the ENSANUT-2018 were used [17]. The variables related to the possession of goods or services are dichotomous, that is, 0 if the household does not possess the good or service and 1 if it does (refrigerator, computer, washing machine, blender, microwave, iron, TV, DVD, heater, telephone line, car or van, internet access, cable TV access). For household characteristics, access to basic services was defined as ordinal variables having between three and four categories each (water, floor, roof, toilet, number of rooms). Each asset was assigned a weight (factor score) generated through principal components analysis, and the resulting asset scores were standardized to a standard normal distribution with a mean of 0 and a standard deviation of 1. Each household was assigned a score for each asset, and the scores for each household were summed.

From the results that included the sample weights, households were classified according to the total household score (continuous variable) and into five equal categories (quintiles): “very poor”, “poor”, “medium”, “rich”, and “very rich” (categorical variable).

#### 2.3.3. Covariables

The following study covariates were used and selected based on previous studies, the PROGRESS framework [18,19], and data available in the ENSANUT-2018. For the assessment of inequalities, the variables used were age group (15 to 19 years, 20 to 29 years, 30 to 39, years, and 40 to 49 years) [7,9,20]; educational level (up to primary, secondary, higher) [6,7]; currently working (no, yes) [21]; ethnicity (“non-indigenous” for individuals who self-reported as afro-ecuatorian, white, mestizo, montubio, or others, and “indigenous” for those who self-reported as indigenous) [22,23]; and, area of residence (rural, urban). The following variables were used for the analysis of associated factors: age group (15 to 19 years, 20 to 29 years, 30 to 39, years, and 40 to 49 years) [7,9,20]; educational level (up to primary, secondary, higher) [21]; marital status (married, cohabiting) [10]; currently working (no, yes); parity (0 to 1, 2, 3 or more children) [24,25]; ethnicity (indigenous, non-indigenous) [23]; health insurance (yes, no) [26]; area of residence (rural, urban) [7]; region of residence (Highlands, Coast, Amazon, Island) [13]; and wealth quintile (“very poor”, “poor”, “medium”, “rich”, and “very rich”) [6,7].

### 2.4. Statistical Analysis

Stata 17.0 (StataCorp, College Station, TX, USA) was used for data analysis. The sample weights and design of the ENSANUT-2018 were included in all estimations using the ‘svy’ command.

A descriptive analysis was performed using absolute and relative frequencies for categorical variables. A bivariate analysis was performed to evaluate differences between the dependent and independent variables using the Chi-square test with Rao–Scott correction.

Concentration curves (CC) and Erreygers concentration indices (ECI) were used to estimate inequality in the use of MCM [27]. The concavity of the CC was taken into account, as well as their position in reference to the equality line (the farther away the CC, the greater the inequality). In addition, when the CC was below the equality line, it was interpreted that the use of MCM had a higher concentration in the population with a higher wealth index; on the contrary, if the CC were above the equality line, the concentration of MCM use would be higher in the population with a lower wealth index. The ECI values range between −1 and 1, where 0 represents equality, a positive value indicates a greater concentration of the outcome of interest in the population with a higher wealth index (pro-rich inequality), while a negative value indicates the opposite (pro-poor inequality) [16].

For the crude and multivariate analysis, generalized linear models of the Poisson family with log link function and sandwich variance were used. In the adjusted model (multivariate), variables that presented a *p*-value < 0.2 in the crude model were included; otherwise, they were excluded from that phase of the analysis. Crude and adjusted PR with 95% CI were calculated, taking into account a *p*-value < 0.05 as statistically significant in the adjusted model.

### 2.5. Ethical Considerations

The *Universidad Científica del Sur* approved the conduct of this study (129-DACMH-DAFCS-U. CIENTÍFICA-2022) and considered that it did not require ethical evaluation. Likewise, since this was a secondary analysis of anonymized data, there was no contact with human beings, and thus, there is no risk or possibility of identifying the survey participants. Similarly, the ENSANUT-2018 interviewers obtained informed consent from the participants prior to the application of the questionnaires. Finally, the databases used in this study are in the public domain and are made available by INEC on its website: https://www.ecuadorencifras.gob.ec/salud-salud-reproductiva-y-nutricion/ (accessed on 14 November 2022).

## 3. Results

The majority of study participants belonged to the 30–39-year age group (40.1%), had a secondary education (44.9%), were cohabitants (52.7%), and were unemployed (48.5%) (Table 1). In addition, the majority had three or more children (44.2%), were non-indigenous women (93.4%), did not have health insurance (68.7%), belonged to the richer wealth quintile (21.5%), were living in the urban area (69.6%), and belonged to the coastal region (51.1%).

The proportion of MCM use in the study population was 92.8%. All the variables included in the study, except age group and region, were statistically significantly associated with the use of MCM. Most of the participants who used MCM belonged to the 30–39 years of age group (40.1%), had a secondary education (45%), and were cohabitants (53.3%) (Table 2). In addition, most did not have health insurance (69.1%) and belonged to the middle wealth index (21.3%).

**Figure 1:**
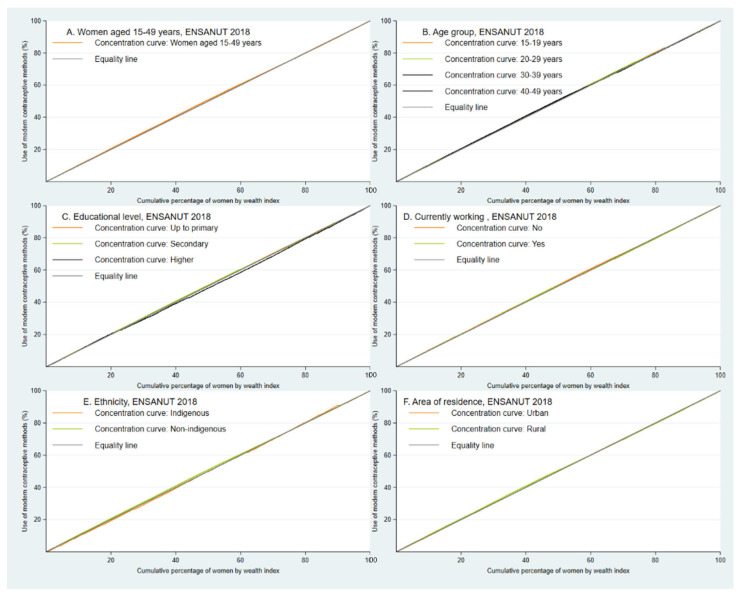
Concentration curves. Estimates include weights and ENSANUT 2018 sample specifications.

In the analysis of the CC, we did not identify a predominant concentration in the use of MCM, either in the population of all women aged 15 to 49 years, or in the evaluation of the CC according to age group, educational level, occupation, ethnicity, and area of residence of the participants (Figure 1).

When estimating the ECI for the variables of interest in the use of MCM, we found that a higher educational level presented a significant pro-rich concentration in the use of MCM. On the other hand, women belonging to the 20–29-year age group, women who did not have a job, and non-indigenous women presented a significant pro-poor concentration in the use of MCM (Table 3).

The frequencies of MCM use were 9%, 7%, and 4% lower in women aged 40–49, 30–39, and 20–29 years, respectively, compared with those aged 15–19 years. Likewise, participants who were currently working had a 3% lower frequency of MCM use compared with those who were not working. Women who lived in urban areas had a lower probability (adjusted PR [aPR]: 0.98; 95% CI: 0.97–0.99) of MCM use compared with those living in rural areas. On the other hand, cohabiting participants had a 2% higher prevalence of MCM use compared to married women. In addition, parity was significantly associated with MCM use; thus, participants with two or three or more children had 5% and 10% higher frequency of use, respectively, than those who had one or fewer children. Women belonging to the highest wealth quintile had a 3% higher frequency of MCM use compared to women in the lowest quintile. Finally, non-indigenous women had a higher probability of using MCM (aPR 1.07) compared to indigenous women (Table 4).

## 4. Discussion

Our study aimed to determine socioeconomic inequalities and factors associated with the use of MCM in the population of sexually active women of childbearing age in Ecuador according to ENSANUT-2018. Pro-rich inequality was identified in the concentration of MCM use in women with higher educational levels. Similar results were observed in a study conducted in 2020, which used information from 11 middle- and low-income countries in the African and Asian regions. The study revealed inequalities in the use of MCM, favoring women with higher wealth and better educational levels [11]. In Ecuador, this could be explained by the fact that almost half of the women participants in our study did not have a job at the time of the survey. Hence, while education levels may be high, the factors of wealth and employment status could still play crucial roles in determining access to MCM. These factors are closely linked to women’s empowerment, a vital aspect influencing decision-making regarding self-care and health-seeking behaviors [28,29]. Therefore, although 9 out of 10 women of the sexually active population of women of childbearing age in Ecuador use MCM, there are still socioeconomic inequalities that could be addressed by promoting the free and extended use of MCM in all the population subgroups.

On the other hand, our analysis identified pro-poor inequalities in the concentration of MCM use according to the categories of age group of 20 to 29 years, women with no work, and non-indigenous women. This pro-poor concentration is contrary to that identified in most previous studies [7,11,30]. However, we could not identify any study elucidating inequalities within the subgroups represented in the present analysis. Concerning this, it could be suggested that women between 20 and 29 years of age living in a disadvantaged situation of wealth could present a greater use of CM, due to fear of becoming pregnant at a very early age, or because they do not yet have job stability. Likewise, the use of MCM could be concentrated in women without work and with a lower level of wealth, because they do not find it viable to become pregnant and support a child due to the lack of their own income in addition to the low level of wealth. Finally, the low level of wealth could also condition the fact that self-identified non-indigenous women are more likely to use MCM than their wealthier counterparts, who may be seeking to become pregnant, or who have sufficient economic solvency to have children, even if unplanned.

Despite the inequalities identified according to some sociodemographic characteristics of the population of women of childbearing age in Ecuador in 2018, notable improvements should be highlighted in relation to the frequency of use of MCM and reduction in inequalities in this population. Thus, in 2004, inequality in the use of MCM in Ecuador was considered intermediate in an analysis of inequalities in 23 countries in LAC [7]. This study showed that there was a difference of 25 percentage points in the use of MCM between the highest and lowest quintile of wealth, being higher in the highest quintile. However, according to our study, this difference is currently 4.2 percentage points, which reflects great progress in access to MCM in recent years in this country. This improvement can be attributed to the health reforms implemented in Ecuador by the Ministry of Public Health in 2014 through the “Regulation to regulate access to contraceptive methods.” The primary goal of this regulation was to provide comprehensive information and counseling regarding the use of MCM while ensuring their free and timely distribution based on the Level of Care, including emergency oral contraception, to the entire population, with a particular focus on adolescents, young adults, and individuals in need of such services. Importantly, there are no prerequisites for receiving these contraceptives, meaning that individuals do not require authorization from family members or partners to access this essential information. As part of this initiative, all first-level healthcare facilities in Ecuador are equipped to provide various MCMs [31].

The factors associated with MCM use were age, marital status, occupation, parity, ethnicity, area of residence, and living on the coast. In the study population, older age groups were associated with a lower probability of using MCM compared to the younger group aged 15 to 19 years. This inverse association of MCM use with age has been reported in previous studies [9,20] and could be related to the fact that most young women are still in school or the first years of university and do not have the economic solvency required to raise a child. Moreover, younger women may be more sexually active, and the frequent pattern of MCM use could be due to a greater information about family planning methods in school campaigns or the media [32,33]. However, it has been reported that, in other contexts, younger women may have a lower frequency of MCM use, due to a lack of information, costs, possible adverse events, or in contexts in which having sex under the age of 18 is prohibited by law [8,34].

We found that there was a higher use of MCM in cohabiting women compared to those who were married. As in our study, an analysis of 73 low- and middle-income countries found that married, childless, adolescent women had a lower prevalence of MCM use [35]. This could be attributed to the fact that women in a cohabiting marital status may be inclined to opt for safer contraceptive methods to prevent unintended pregnancies, possibly due to feeling socially disadvantaged or a sense of social responsibility [36]. On the contrary, those who are married consider the possibility of having more children during the marriage, and for this reason 16.8% of married women do not use MCM [37]. In addition, it has been reported that married women do not use MCM because of their partners’ influence (64.2%), since some partners wish to have more children or believe that the use of a CM allows women more freedom to be unfaithful because they cannot become pregnant [38]. In addition, there are those who oppose the use of CM for religious reasons. Ninety-two percent of Ecuadorians have some type of religious affiliation, with the largest percentage being Catholic. The leaders of the Catholic Church do not agree with the laws that promote the free delivery of MCM, so many Catholics choose not to use it [38,39].

In Ecuador, women without work were more likely to use MCM than those who were currently working. In this regard, although it would be expected that occupation may represent a higher socioeconomic level and, therefore, be associated with greater use of MCM, other characteristics could potentially modify this association. For example, a study based on the National Demographic and Health Survey (ENDSA) in Bolivia found that women who worked but were not professionals were 75% more likely not to use MCM [40]. In this sense, the level of education of the woman could be an important modifying factor in the association identified. Likewise, work may limit the use of MCM, possibly because women prioritize their work and have scarce time to go to a health facility to receive information on family planning and contraception [40].

A higher prevalence of MCM use was found in women with two or three or more children compared to those with one or no children. A study conducted in Sao Paulo, Brazil, indicated that women with two or more children considered it important to prevent pregnancy and not to have more children [24]. These findings are consistent with those of a Peruvian study in which women without children make less use of MCM compared to those who had more than one child [25]. This is because women who already have children use CM because they are satisfied with parity and do not plan to have a new pregnancy. For this reason, it is important to promote family planning and take into account the feeling of satisfaction that women have in relation to motherhood, their autonomy, professional development, and participation in society; furthermore, they have the option to choose to use MCM to plan their next pregnancy if they wish to do so.

The use of MCM was higher in non-indigenous women compared to indigenous women, similar to the results of the *Encuesta Nacional de la Dinámica Demográfica de México* 2018 (ENADID), in which 83.4% of indigenous language speakers had no desire to plan or space their pregnancies [41]. This could explain the lower use of MCM among indigenous women, which may not be directly related to belonging or not to any ethnic group, but rather to several related factors including the place of residence, education, economic participation, age, and having had children [41]. The high fertility rate among the indigenous population and the low prevalence of MCM use, in addition to the high maternal mortality indicators, place indigenous women as a priority group for sexual and reproductive health care, making it important to encourage family planning education in the less accessible areas where these population groups live.

Another relevant finding was that residents of urban areas had lower use of MCM. This result is similar to a study carried out in Costa Rica showing that residents of urban areas were less likely to use MCM than those in rural areas [42]. However, most studies show a different pattern, with urban areas having a higher prevalence of CM use [43,44,45,46,47]. Explanations about these findings are scarce in the literature, and although the urban–rural differences in the frequency of MCM use are not comprehensive in Ecuador, it is necessary to deepen research on the topic in order to have a better understanding of this pattern, which seems contrary to the majority of countries.

We found that living in the coastal region was associated with a lower probability of using MCM compared to living in the highlands in Ecuador. In contrast, in Peru (2021) the use of MCM was higher among women residing on the coast (60.3%) or in the jungle (54.6%), compared to the highlands (50.7%) [48]. This difference between the results of both countries is due to the geographic location of their capitals, with Lima located in the coastal region of Peru, and Quito in the highlands region of Ecuador. On the other hand, family planning programs, the benefits of the modernization process of society, and its influence on behavioral values about fertility could have a greater impact in each of the main cities of the country. Accordingly, in the countries involved, the management of sexual and reproductive health resources and programs is centralized, favoring people living in the main regions of each country. Therefore, coverage and access to health services should be universal and decentralized, with emphasis on the promotion and prevention of reproductive health.

The primary limitation of this study was the utilization of a yes-or-no variable to assess contraceptive use, which encompassed a range of contraceptive options with varying degrees of effectiveness. The list included highly effective contraceptive choices as well as less effective ones like condoms, and even emergency contraception. Consequently, drawing specific conclusions about the usage of a particular contraceptive method becomes challenging. Additionally, the available data did not permit the differentiation of long-term users from new users, which may have allowed us to evaluate the problem from another angle. It is also worth noting that not all women use hormonal birth control pills to prevent pregnancy, but they can be used medicinally for acne, menstrual disorders, or other reasons. Furthermore, despite the availability of vaginal rings in the public health system in Ecuador, it appears that the survey’s definition of MCM did not explore the utilization of these methods [49]. Another important limitation of this study is its cross-sectional design, making it impossible to establish cause-and-effect relationships among the factors evaluated and the use of MCM. Additionally, there may also be a social desirability bias, as some respondents may lie about the use of MCM out of embarrassment or to seek approval from research observers.

Despite these limitations, this study uses a nationally representative database from the ENSANUT with the most up-to-date information possible. Taking this into account, the information reported could be useful for decision-making, public policy formulation, and monitoring of the state of MCM use, its associated factors, and inequalities in Ecuador.

## 5. Conclusions

In the population of sexually active women of childbearing age in Ecuador, pro-rich inequalities were identified in the concentration of MCM use among women with a higher education. Likewise, pro-poor inequalities were identified in the concentration of MCM use according to the age group of 20 to 29 years, women with no occupation, and non-indigenous women. On the other hand, the factors associated with the use of MCM in the study population were age group, marital status, occupation, parity, ethnicity, area of residence, and living on the coast. Although important improvements in inequalities in the use of MCM in women of childbearing age in Ecuador were evidenced, there are persistent inequalities in some specific population groups. It is necessary to design promotion and education campaigns on the use of MCM focused on providing the necessary information for more responsible and effective use, expanding coverage and access to universal and decentralized health services. It is also necessary to achieve greater awareness of the partners of women of childbearing age in sexual and reproductive health, so that they respect the decisions that women make regarding the use of contraceptive methods.

## Figures and Tables

**Table 1:** Characteristics of the participants included in the study (*n* = 19,106).

Characteristics	*n*	% *
Age group (years)		
15–19	934	3.9
20–29	6731	29.4
30–39	7261	40.1
40–49	4180	26.6
Educational level		
Up to primary	6390	33.7
Secondary	8801	44.9
Higher	3915	21.4
Marital status		
Married	8815	47.3
Cohabitant	10,291	52.7
Currently working		
No	10,135	51.5
Yes	8971	48.5
Parity		
0–1	4905	24.5
2	5914	31.3
3 or more	8287	44.2
Ethnicity		
Indigenous	2127	6.6
Non-indigenous	16,979	93.4
Health insurance		
Yes	5442	31.3
No	13,664	68.7
Wealth quintile		
Very poor	3984	16.2
Poor	4128	20.5
Medium	4102	21.2
Rich	3772	21.5
Very rich	3120	20.6
Area of residence		
Rural	7144	30.1
Urban	11,962	69.9
Region of residence		
Highlands	7216	43.5
Coast	7285	51.1
Amazon	3908	5.2
Island	697	0.2

* Estimates include the weights and ENSANUT-2018 sample specifications.

**Table 2:** Characteristics of the participants according to the use of modern contraceptive methods.

Characteristics	MCM		*p* Value *
	No (*n* = 1389)	Yes (*n* = 17,717)	
	% (95% CI)	% (95% CI)	
Overall	7.2 (6.6–7.9)	92.8 (92.1–93.4)	
Age group (years)			
15–19	2.2 (1.3–3.8)	4.0 (3.6–4.4)	0.131
20–29	27.8 (24.2–31.6)	29.5 (28.4–30.7)	
30–39	40.6 (36.1–45.3)	40.1 (38.8–41.4)	
40–49	29.4 (25.0–34.2)	26.4 (25.2–27.6)	
Educational level			
Up to primary	26.3 (22.4–30.7)	34.3 (32.8–35.8)	<0.001
Secondary	43.3 (38.8–47.8)	45.0 (43.5–46.5)	
Higher	30.4 (26.1–35.1)	20.7 (19.5–22.0)	
Marital status			
Married	55.5 (50.6–60.3)	46.7 (45.2–48.1)	0.001
Cohabitant	44.5 (39.7–49.4)	53.3 (51.9–54.8)	
Currently working			
No	38.6 (34.2–43.2)	52.5 (51.1–54.0)	<0.001
Yes	61.4 (56.8–65.8)	47.5 (46.0–48.9)	
Parity			
0–1	35.9 (31.3–40.8)	23.6 (22.6–24.7)	<0.001
2	34.6 (30.4–39.0)	31.0 (29.9–32.2)	
3 or more	29.5 (25.6–33.8)	45.3 (44.1–46.6)	
Ethnicity			
Indigenous	11.1 (8.2–14.8)	6.2 (5.6–6.9)	<0.001
Non-indigenous	88.9 (85.2–91.8)	93.8 (93.1–94.4)	
Health insurance			
Yes	36.1 (31.6–40.8)	30.9 (29.5–32.4)	0.027
No	63.9 (59.2–68.4)	69.1 (67.6–70.5)	
Wealth quintile			
Very poor	12.3 (9.8–15.3)	16.5 (15.4–17.7)	<0.001
Poor	16.2 (13.0–20.0)	20.8 (19.7–22.0)	
Medium	20.3 (17.1–23.9)	21.3 (20.1–22.5)	
Rich	32.1 (27.6–37.0)	20.7 (19.5–21.9)	
Very rich	19.1 (15.8–23.0)	20.7 (19.2–22.3)	
Area of residence			
Rural	22.6 (19.6–26.0)	30.7 (29.4–32.0)	<0.001
Urban	77.4 (74.0–80.4)	69.3 (68.0–70.6)	
Region of residence			
Highlands	43 (38.2–47.8)	43.5 (41.9–45.2)	0.667
Coast	51.1 (46.4–55.9)	51.1 (49.5–52.7)	
Amazon	5.6 (4.7–6.6)	5.2 (4.9–5.5)	
Island	0.3 (0.3–0.4)	0.2 (0.2–0.2)	

MCM: modern contraceptive methods; CI: confidence interval. Estimates include the weights and ENSANUT 2018 sample specifications. * The *p*-value was calculated using the Rao-Scott Chi-squared test.

**Table 3:** Use of modern contraceptive methods according to concentration indices in the population of women aged 15 to 49 years in Ecuador, 2018.

Characteristics	MCM			
	ECI	SE	*p* Value *	*p* Value across Categories **
Overall	−0.02393248	0.00707563	0.0007	
Age groups (years)				
15–19	−0.01827511	0.02435466	0.453	0.027
20–29	−0.02732495	0.01230476	0.027	
30–39	−0.01934179	0.01065013	0.070	
40–49	−0.0146222	0.01381501	0.290	
Educational level				
Up to primary	−0.01697803	0.01093103	0.121	0.0514
Secondary	−0.01367809	0.01017993	0.179	
Higher	0.05022723	0.01725384	0.004	
Currently working				
No	−0.02500584	0.00858693	0.004	0.0769
Yes	0.00051558	0.0115945	0.965	
Ethnicity				
Indigenous	0.01719356	0.03613912	0.634	0.1899
Non indigenous	−0.03107635	0.00706549	<0.001	
Area of residence				
Rural	−0.00125461	0.00936669	0.8935	0.258
Urban	−0.01627268	0.00940888	0.084	

Estimates include the weights and ENSANUT-2018 sample specifications. SE: Standard error. ECI: Erreygers concentration index. MCM: modern contraceptive methods. * F-test. ** z-test for comparing two categories and F-test for comparing three or more categories.

**Table 4:** Factors associated with the use of modern contraceptive methods in the population of women aged 15 to 49 years in Ecuador, 2018.

Characteristics	Crude Model		Adjusted Model *	
	PR (95% CI)	*p* Value	aPR (95% CI)	*p* Value
Age group (years)				
15–19	Reference		Reference	
20–29	0.97 (0.95–1.00)	0.028	0.96 (0.93–0.99)	0.003
30–39	0.97 (0.94–0.99)	0.012	0.93 (0.89–0.96)	<0.001
40–49	0.96 (0.93–0.99)	0.004	0.91 (0.88–0.95)	<0.001
Educational level				
Up to primary	Reference		Reference	
Secondary	0.99 (0.97–1.00)	0.053	1.00 (0.98–1.01)	0.707
Higher	0.95 (0.93–0.97)	<0.001	0.98 (0.95–1.01)	0.116
Marital status				
Married	Reference		Reference	
Cohabitant	1.03 (1.01–1.04)	0.001	1.02 (1.00–1.04)	0.027
Currently working				
No	Reference		Reference	
Yes	0.96 (0.95–0.97)	<0.001	0.97 (0.96–0.99)	<0.001
Parity				
0–1	Reference		Reference	
2	1.03 (1.01–1.05)	0.011	1.05 (1.02–1.08)	<0.001
3 or more	1.06 (1.04–1.09)	<0.001	1.10 (1.07–1.13)	<0.001
Ethnicity				
Indigenous	Reference		Reference	
Non-indigenous	1.06 (1.02–1.10)	0.002	1.07 (1.03–1.12)	0.001
Health insurance				
Yes	Reference		Reference	
No	1.02 (1.00–1.03)	0.036	1.00 (0.98–1.02)	0.838
Wealth quintile				
Very poor	Reference		Reference	
Poor	1.00 (0.98–1.02)	0.785	1.01 (0.99–1.03)	0.513
Medium	0.98 (0.97–1.00)	0.112	1.01 (0.98–1.03)	0.617
Rich	0.94 (0.92–0.97)	<0.001	0.97 (0.95–1.00)	0.054
Very rich	0.99 (0.97–1.01)	0.192	1.03 (1.00–1.06)	0.036
Area of residence				
Rural	Reference		Reference	
Urban	0.97 (0.96–0.99)	<0.001	0.98 (0.97–0.99)	0.006
Region of residence				
Highlands	Reference		Reference	
Coast	1.00 (0.98–1.01)	0.888	0.98 (0.96–0.99)	0.034
Amazon	0.99 (0.98–1.01)	0.438	0.99 (0.97–1.01)	0.207
Island	0.96 (0.94–0.99)	0.010	0.98 (0.95–1.00)	0.094

PR: prevalence ratio; aPR: adjusted prevalence ratio; CI: confidence interval. * Adjusted for all the variables shown in the column. Estimates include the weights and ENSANUT-2018 sample specifications.

## Data Availability

The databases, glossaries of terms and materials used in this study are in the public domain and are made available by the National Institute of Statistics and Census (INEC) and can be consulted at: https://www.ecuadorencifras.gob.ec/salud-salud-reproductiva-y-nutricion/ (accessed on 14 November 2022).
